# Optimization of Screening Media to Improve Antimicrobial Biodiscovery from Soils in Undergraduate/Citizen Science Research-Engaged Initiatives

**DOI:** 10.3390/antibiotics13100956

**Published:** 2024-10-11

**Authors:** Leah McPhillips, John O’Callaghan, Carmel Shortiss, Stephen A. Jackson, Niall D. O’Leary

**Affiliations:** 1 John Innes Centre, Norwich NR4 7UH, UK; leah.mcphillips@jic.ac.uk; 2 School of Microbiology, University College Cork, T12 K8AF Cork, Ireland; j.ocallaghan@ucc.ie (J.O.); c.shortiss@ucc.ie (C.S.); sjackson@ucc.ie (S.A.J.); 3 Environmental Research Institute, University College Cork, T12 K8AF Cork, Ireland

**Keywords:** biodiscovery, antimicrobial resistance, screening media, soil extract agar, N-acetyl glucosamine, actinobacteria, *Streptomyces*

## Abstract

**Background/Objectives:** Research-engaged academic institutions offer the opportunity to couple undergraduate education/citizen science projects with antimicrobial biodiscovery research. Several initiatives reflecting this ethos have been reported internationally (e.g., Small World, Tiny Earth, MicroMundo, Antibiotics Unearthed). These programs target soil habitats due to their high microbial diversity and promote initial screening with non-selective, nutrient media such as tryptic soy agar (TSA). However, evaluation of published outputs to date indicates that isolate recovery on TSA is consistently dominated by the genera *Bacillus*, *Pseudomonas,* and *Paenibacillus*. In this study, we evaluated the potential of soil extract agar to enhance soil isolate diversity and antibiosis induction outcomes in our undergraduate Antibiotics Unearthed research program. **Methods:** We comparatively screened 229 isolates from woodland and garden soil samples on both tryptic soy agar (TSA) and soil extract agar (SEA) for antimicrobial activity against a panel of clinically relevant microbial pathogens. **Results:** On one or both media, 15 isolates were found to produce zones of clearing against respective pathogens. 16S rRNA gene sequencing linked the isolates with three genera: *Streptomyces* (7), *Paenibacillus* (6), and *Pseudomonas* (2). Six of the *Streptomyces* isolates and one *Pseudomonas* demonstrated antimicrobial activity when screened on SEA, with no activity on TSA. Furthermore, incorporation of the known secondary metabolite inducer N acetyl-glucosamine (20 mM) into SEA media altered the pathogen inhibition profiles of 14 isolates and resulted in broad-spectrum activity of one *Streptomyces* isolate, not observed on SEA alone. In conclusion, SEA was found to expand the diversity of culturable isolates from soil and specifically enhanced the recovery of members of the genus *Streptomyces*. SEA was also found to be a superior media for antibiosis induction among *Streptomyces* isolates when compared to TSA. It was noted that *Paenibacillus* isolates’ antibiosis induction demonstrated a strain-specific response with respect to the growth media used. **Conclusions:** The authors propose SEA inclusion of in soil screening protocols as a cost-effective, complementary strategy to greatly enhance outcomes in undergraduate/citizen science-engaged antimicrobial biodiscovery initiatives.

## 1. Introduction

The emergence of the post-antibiotic era is a critical global health threat, with antimicrobial resistance (AMR) infection-related deaths projected to reach 10 million p.a. by 2050 [1]. The threat is compounded by declining private sector investment in biodiscovery due to high costs, long market lead times (10–15 years), and low percentage success rates [1,2]. One response to this challenge has been the development of initiatives for undergraduate engagement in the exploration of soil ecosystems for antimicrobial compound-producing bacteria. The Small World Initiative (Yale), later consolidated as Tiny Earth (University of Wisconsin), has become an internationally influential program in this respect [3,4]. In addition to supporting biodiscovery, such engagement in real-world, global health-related research can increase student retention in science-based academic programs. Several groups have also incorporated elements of citizen science into derivatives of Tiny Earth to promote public awareness of AMR issues [5,6,7,8,9].

The Tiny Earth methodology comprised three stages, which have been well described previously [3] and widely disseminated internationally through educator training workshops hosted by Tiny Earth [3]. In brief, stage 1 isolation protocols promote the use of nutritionally-replete or minimally-selective agar, such as tryptic soy agar and potato dextrose agar, to encourage the growth of a wide diversity of soil microbes. Screening of isolates for antimicrobial activity in stage 2 involves overlays with representatives of the ESKAPE pathogens, *Enterococcus faecium*, *Staphylococcus aureus*, *Klebsiella pneumoniae*, *Acinetobacter baumannii*, *Pseudomonas aeruginosa*, and *Enterobacter*, respectively. These pathogens pose a significant threat to human health due to their rapid and diverse acquirement of resistance to antibiotics, as well as their ability to thrive in healthcare settings [10,11]. Variations on this approach have also reported successful screening for antifungal activities from soil isolates [12]. Stage 3 combines liquid cultivation and compound extraction approaches with the potential molecular identification of isolates via 16S rRNA gene amplification and sequencing [3]. In recent years, the incorporation of advanced downstream technological approaches (e.g., metabolomics, genomics, and chemical analyses) to maximize the potential for genuine discovery and natural product development from these initiatives has been seen [4,13,14]. However, given the educational setting and/or citizen science nature of many studies, the research infrastructure and technical capacity required to deliver on stage 3 and beyond can become limiting. It has been suggested, therefore, that the crowdsourcing element of these initiatives should focus on maximizing the recovery and antibiosis induction of soil isolates from niche habitats, with downstream characterization devolved to specialist laboratories [15].

The School of Microbiology at University College Cork has been affiliated with Tiny Earth since 2015, when the initiative was promoted in the UK and Ireland under the auspices of the Microbiology Society as “Antibiotics Unearthed” [7]. Early iterations of the program presented challenges in achieving pure cultures as TSA nutritional media selected for fast-growing, spreading colonies. Low isolate diversity was also an issue with the frequent dominance of isolates by members of the Bacillales and Pseudomonadales orders. Examination of recently published studies suggested similar outcomes among other groups. Pino-Hurtado and co-workers identified 52 bioactive compound-producing bacteria among 1220 soil isolates. However, 67% and 14% of the antibiotic producers were derived from genera within the orders Bacillales and Pseudomonadales, respectively [12]. Only one *Streptomyces* isolate was reported in the study despite soil being a common source of members of this genus. Fernández-Fernández et al. reported similar outcomes in a screen of 2600 soil isolates, where the most frequent genera among the 132 isolates demonstrating antimicrobial activity were *Bacillus*, followed by *Pseudomonas* and *Paenibacillus* [9]. No *Streptomyces* isolates were reported. These observations are not a criticism of the initiative approach. Indeed, one of the express aims of Tiny Earth is to explore “side-lined” microbial genera for novel compounds [3]. However, *Streptomyces* species produce approximately half of all antibiotics that are in clinical use today [2]. Furthermore, whole genome sequencing has revived interest in filamentous Acintomycetes following the identification of antimicrobial biosynthetic gene clusters (BCGs) that are not expressed under routine laboratory cultivation conditions [2]. Therefore, while TSA application in Tiny Earth protocols may support certain bacterial taxa, it is not optimal for the recovery of slower-growing actinomycetes or the induction of secondary metabolism [14].

De Groot and co-workers reported the recovery of 18 *Streptomyces* isolates with antimicrobial activity from a bank of 390 soil isolates when TSA was replaced with Actinomycete Isolation Agar and R2A media with nalidixic acid/cycloheximide to exclude fungal and Gram-negative bacterial growth [14]. While this approach enhanced actinobacterial isolation, such synthetic media may introduce cost barriers to high-volume application in citizen science or undergraduate settings. In contrast, Hamaki and colleagues have previously reported the successful use of a low-cost soil extract agar to recover a range of soil bacteria, including novel actinomycete representatives, based on mimicking natural ecosystem conditions [16] The incorporation of such media into the stage 1 phase of Tiny Earth-based initiatives could offer a cost-effective enrichment step for actinomycete recovery. Being derived from soils, such media could also provide unique environmental cues required for secondary metabolism and antibiosis induction. These may include ecosystem-related nutritional stress conditions and/or the presence of biological compounds with signalling effects. For example, N-acetylglucosamine (Glc-NAc) is a component of peptidoglycan in bacteria and chitin in fungi, which has been found to induce secondary metabolism in some *Streptomyces* species when incorporated into media [17,18].

In this study, we sought to explore the potential for soil extract agar to enhance the isolation and induction of the antimicrobial activity of actinobacteria from soil samples in a modification of the Tiny Earth protocol. We also investigated whether the incorporation of GlcNAc altered isolate antibiosis profiles against ESKAPE pathogens. SEA was found to enrich for *Streptomyces* species but did not selectively exclude isolates from other genera such as *Paenibacillus* and *Pseudomonas*. SEA was observed to be essential for the induction of antibiosis among the *Streptomyces* isolates, irrespective of species type. In contrast, SEA appeared to affect the antimicrobial activity of *Paenibacillus* in a species-specific manner, inhibiting antibiosis by *Paenibacillus polymyxa* but not *Paenibacillus peoriae*. Glc-NAc incorporation into SEA media affected the antibiosis profiles of 14 out of the 15 isolates, both positively and negatively. One *Streptomyces* isolate was found to exhibit broad-spectrum inhibition only when grown on SEA containing Glc-NAc. Implications for the enhancement of crowdsourcing contributions to antibiotic discovery pipelines are discussed.

## 2. Results

### 2.1. Initial Isolation and Screening of Soil Bacteria for Antibacterial Activity

Using an array of isolation media (TSA, Starch-casein-nitrate, SEA, and SEA + cycloheximide), 229 microbial isolates were recovered from woodland and garden soil samples. Initial screening for antimicrobial activity against ESKAPE pathogens was conducted on the same media on which each isolate was recovered. In total, 15 isolates were found to exhibit antimicrobial activity and were identified by 16S rRNA gene sequencing (>98% identity) as belonging to three genera: *Streptomyces* (7/15, 46%), *Paenibacillus* (6/15, 40%) and *Pseudomonas* (2/15, 13%) (Table 1). The authors noted that 12/15 (80%) of the isolates demonstrating antibiosis were isolated on SEA media, with the remainder (including two *Streptomyces atratus* strains) isolated on starch casein nitrate agar. While *Streptomyces* were the most abundant species recovered, they were closely followed by *Paenibacillus* and *Pseudomonas* species (Table 1). The findings suggest that SEA media (under the conditions tested), offered significant potential to increase the actinobacteria abundance among isolates recovered from soil without excluding other desirable or commonly recovered taxa reported by other groups using TSA [9,12,19].

### 2.2. Comparative Evaluation of TSA and SEA for the Induction of Antimicrobial Activity

In early iterations of our Antibiotics Unearthed program with students, the initial screening of isolates was conducted on TSA. Therefore, we investigated whether TSA of SEA media produced differing induction of antibiosis among the 15 isolates of interest. The antibiosis profiles of isolates transferred to TSA plates and overlaid with ESKAPE pathogens are presented in Table 2, with a comparative induction on SEA reported in Table 3. In both tables, relative antimicrobial activity was quantified as diameters of observed zones of clearing. As shown in Table 2, the use of TSA as the growth media was observed to support broad-spectrum antimicrobial activity among *Paenibacillus* isolates, with one *P. peoriae* isolate inhibiting all six ESKAPE pathogens. Some variance was observed in antibiosis profiles between *Paenibacillus* species on TSA, whereby *P. polymyxa* isolates did not inhibit *A. johnsonii* DSM 6963, while *P.* sp. Strain Y2 and *P. peoriae* isolates strongly inhibited this pathogen. However, when plated on SEA as the antibiosis screening substrate *P. polymyxa* isolates exhibited the inhibition of *A. johnsonii* DSM 6963 comparable to *P. peoriae* species (Table 3). The *Pseudomonas* isolates were also found to exhibit altered antibiosis profiles based on the growth media. *Pseudomonas azotoformans* only exhibited the inhibition of *S. aureus* N670949 when cultivated on SEA (Table 3), while for *Pseudmonas jessenii,* broad-spectrum antibiosis was only maintained on TSA (Table 2). Therefore, while the specific mechanism has yet to be elucidated, the data suggest the complementary inclusion of SEA media in antibiosis screening may expand the range of inhibition induced among *Paenibacillus* and *Pseudomonas* species, which are commonly isolated during Tiny Earth-related programs [12,14,19]. With respect to the *Streptomyces* isolates in this study, the growth media appeared to be critical in antibiosis induction. As shown in Table 2, screening on TSA did not induce antibiosis in the majority of *Streptomyces*, with only a weak inhibition of *E. faecium* NCIMB 11508 observed with one isolate. In contrast, six of the seven *Streptomyces* isolates exhibited antibiosis against one or more ESAKPE pathogens when screened on SEA (Table 3). The inhibition of *S. aureus* was most common (4/6, 75%) among *Streptomyces* isolates, while *S. avidnii* inhibited *A. johnsonii* alone. Only the *Streptomyces/Kitosatospora* sp isolate demonstrated broader spectrum activity, inhibiting *E. faecium* NCIMB 11508 and *S. aureus* N 670949. The data presented here strongly support the inclusion of SEA media in antibiosis screening, where actinomycete isolation and induction represent an experimental objective.

### 2.3. Impact of N-Acetyl Glucosamine on Antibiosis Profiles of Isolates

The addition of the signaling molecule N-acetylglucosamine (20 mM) to SEA media produced a range of impacts on observed antibiosis profiles among 14/15 of the soil isolates when compared to screening on SEA alone (Table 4). Most notably, *Streptomyces avidnii* exhibited a broad-spectrum inhibition of all ESAKPE pathogens except *P. aeruginosa* in the presence of GlcNAc, compared to the sole inhibition of *A. johnsonii* DSM 6963 on SEA alone (Table 3). It was also noted that five of the seven *Streptomyces* isolates exhibited the inhibition of *E. faecium* NCIMB 11508 in the presence of GlcNAc, which was not observed on SEA. However, in the case of *S. atratus* and *Kitosatospora,* this coincided with the loss of *S. aureus* inhibition as reported on SEA media (Table 3 and Table 4). *S. goshikiensis* was also found to inhibit *E. aerogenes* NCIMB 101PO2 only in the presence of GlcNAc. The impact of GlCNac on *Paenabacillus* and *Pseudmonas* isolates was more subtle. The broad-spectrum inhibition profile of *P. jensenii* observed on TSA (Table 2), which was eliminated on SEA, was restored on SEA + GlcNAc. In contrast, *P. azotoformans* lost the capacity to inhibit *S. aureus* N670949 in the presence of GlcNAc, compared to SEA alone (Table 3 and Table 4). In relation to *Paenibacillus*, GlcNAc inclusion of in SEA media appeared to enhance the induction of *E. aerogenes* inhibition among *P. polymyxa*. More significantly, however, no inhibition of *P. aeruginosa PA01* by *P. polymyxa* or *P. peoriae* was retained in the presence of GlcNAc compared to TSA and SEA (Table 2 and Table 3). The authors noted that *P. aerugionsa* overlays on SEA + GlcNAc developed a blue/green hue. A review of the literature identified a link between GlcNAc and the expression of pyocyanin, a potent, broad-spectrum antimicrobial resistance factor in *P. aeruginosa* [20,21]. This may explain the loss of antibiosis observed and highlights that cultivation conditions can also alter the sensitivity/physiology of target pathogens, such that screening media outcomes should be interpreted carefully. That being stated, the data suggest that for *Streptomyces* isolates, signaling molecule incorporation into the SEA media may expand the expression of cryptic BCGs and improve biodiscovery outcomes.

## 3. Discussion

2025 will mark the 10th year of our operation of antimicrobial biodiscovery from soil in the School of Microbiology undergraduate program at University College Cork. In the first 3 years combined (2015–2017), we conducted 16S rRNA gene sequencing of a total of 153 isolates with bioactive compound potential, of which only 5 (2%) were identified as members of the genus *Streptomyces.* In the last 3 years (2021–2023), a total of 330 isolates of interest were subjected to 16S identification, with 105 (32%) coming from the genus *Streptomyces.* We attribute a significant degree of this increase in actinobacteria recovery to protocol modifications involving soil extract agar incorporation into the isolation and screening phase. In this study, we applied this approach to quantitatively assess the antibiosis induction of soil isolates on TSA, SEA, and SEA with the signaling molecule N-acetylglucosamine added. Among 229 isolates, 15 were found to demonstrate antimicrobial activity and were selected for the comparative induction study. The data presented in Table 2 and Table 3 demonstrate that SEA was essential for antibiosis induction among *Streptomyces* isolates, which would not have been recovered or given any significance if this media was not included in the soil screening protocol. Our observations are further supported by the results of several other studies applying the Tiny Earth biodiscovery method, where actinobacterial “hits” are typically limited or absent among isolates demonstrating antibiosis on TSA [6,12,14,19]. In light of the resurgence of interest in actinobacteria for antimicrobial peptide synthesis and potential cryptic BGCs, the capacity to enhance their recovery in soil isolation crowdsourcing initiatives is a valuable outcome [1,22,23,24,25].

An additional benefit of SEA, as demonstrated in Table 2 and Table 3, is that it does not selectively exclude other important microbial taxa such as *Paenibacillus* and *Pseudomonas,* and indeed, can induce antibiosis activity in these species when compared to TSA (Table 2 and Table 3). SEA, therefore, provides for greater heterogeneity in the isolation and induction of soil isolates than TSA alone or selective agars, which only target actinobacteria [14]. The authors believe this is a key finding of this study as the aim of the Tiny Earth initiative and international derivatives is to recover and screen as diverse a community as possible from respective soil samples [3]. The mechanism of induction on SEA was not investigated in this study but likely involves nutritional stress-related secondary metabolite production [18,26]. The authors did, however, consider whether the SEA prepared in the lab represented a unique media based on the garden soil type used. To test this hypothesis, SEA was also prepared with (a) commercial compost and (b) commercial clay, and the resulting SEA media was used in repeated antibiosis induction with a subset of six isolates. No qualitative or quantitative difference in the antibiosis profiles of these isolates was observed across the garden-, compost- or clay soil-based SEA media.

The incorporation of N-acetyl glucosamine into SEA was found to alter the antibiosis profiles of the majority of isolates, with *Streptomyces* species being the most affected. Most notably, *S. avidnii* developed broad-spectrum antibiosis against ESKAPE pathogens, which was not observed on SEA (Table 4). These observations align with previously reported impacts of GlcNAc on antibiotic synthesis by Streptomyces involving the induction of sporulation and expression of cryptic BGCs [17,18,26]. Further work is required to evaluate the mechanism of altered antibiosis observed among the isolates in our study. It was also noted that the inhibition of *P. aeruginosa* PA01 was not achieved by any isolate screened on SEA + GlcNAc (Table 4). However, the authors posit that this observation reflects pyocyanin production by *P. aeruginosa* when exposed to acetylglucosamine and its accompanying broad-spectrum antibiotic resistance [20]. Therefore, while GlcNAc may enhance biodiscovery outcomes in isolate screening, its potential impact on the physiology of target organisms should be considered when interpreting antibiosis profiles.

In conclusion, student-sourced antimicrobial biodiscovery initiatives represent dual opportunities for inspirational STEM engagement and genuine research outputs with potential societal impacts. Multiple international studies have demonstrated the capacity for such initiatives to positively impact STEM student recruitment and retention, as well as raising public awareness of AMR as a global health issue [3,4,5,6,7,8,9,12,13,14,15,19,27]. It is entirely feasible that such initiatives may one day deliver a clinically relevant antimicrobial compound, and every effort should be made to optimize this potential. In this study, we focused on optimizing initial screening media in the Tiny Earth protocol, demonstrating that SEA and N-acetylglucosamine inclusion increased the heterogeneity of taxa recovered and enhanced the induction of antibiosis among isolates.

## 4. Materials and Methods

### 4.1. Bacterial Strains and Soil Samples

Representative ESKAPE pathogen cultures were reconstituted on TSA from −80 °C freezer stocks maintained in the School of Microbiology UCC and included *Enterococcus faecium* NCIMB 11508, *Staphylococcus aureus* N670949, *Klebsiella pneumoniae* NCIMB 113218, *Acinetobacter johnsonii* DSM6963, *Pseudomonas aeruginosa* PA01, and *Enterobacter aerogenes* NCIMB 101P2. Potential antibiotic-producing bacteria were isolated from soil samples in the following manner: Samples were obtained from the surrounding soil of four different trees from the genera *Quercus* (oak), *Ulmus* (elm), *Fagus* (beech), and *Acer* (maple/acer), with a soil pH of 5.63, 5.73, 5.50, and 5.39, respectively. The oak tree soil samples were collected from 51.8943° N, 8.5178° W in a grasslands area, while the remaining three were collected from 51.8895° N, 8.6208° W in a woodland area, all in the County Cork region of the Republic of Ireland in early summer. Trees were identified based on leaf structure, and soil samples were collected in duplicate approximately 2 m apart by first removing the surface debris of the soil using a sterilized spatula. The spatula was then resterilized with 70% ethanol, and the soil sample was obtained from the upper soil horizon at a depth of approximately 5–10 cm and placed in a sterile container.

### 4.2. Cultivation Media Preparation

Soil extract agar (SEA) was prepared using a 1:5 ratio of garden soil purchased from a local garden center (10 Kg bag) added to deionized water (dH_2_O), which was mixed well and allowed to sit for approximately 1.5 h, stirring occasionally. This mixture was then filtered to remove large debris, and the filtrate was centrifuged at 7500 revolutions per minute (RPM) for 20 min at 16 °C. The supernatant was then separated, and each per liter of supernatant, 1 g of glucose, 0.2 g of potassium phosphate dibasic, and 15 g of agar were added. The same recipe was used for both the compost- and clay-based SEA, but with either commercial compost or clay soil used to replace garden soil. SEA with 20 mM of N-acetyl-D-glucosamine (D-GlcNAc) was made following the above recipe with the garden soil. D-GlcNAc (Sigma-Aldrich^®^, St. Louis, MO, USA) was dissolved in dH_2_O at a 1:4 ratio, stirring continuously over low heat. This D-GlcNAc solution was filter sterilized using a 0.45 µm membrane filter and added to autoclaved SEA, which had been allowed to cool slightly, to make a 20 mM D-GlcNAc SEA medium. For the SEA + cycloheximide, the SEA was prepared as described above, and the fungicide cycloheximide was added after autoclaving to deselect for fungi and potentially select for antibiotic-producing bacteria. *Streptomyces* agar was made using 27.3 g/L of malt extract broth, 4 g/L of yeast extract, 4 g/L of glucose, 2 g/L of calcium carbonate, and 12 g/L of agar. The Starch Casein Nitrate (SCN) agar consisted of 15 g/L of agar, 0.02 g/L of calcium carbonate, 2 g/L of potassium nitrate, 2 g/L of potassium phosphate dibasic, 2 g/L of sodium chloride, 10 g/L of soluble starch, 0.3 g/L of casein, 0.01 g/L of ferrous sulfate·7H_2_O and 0.05 g/L of magnesium sulfate·7H_2_O, with the latter two being added after autoclaving. The Tryptic Soy Agar (TSA) and Tryptic Soy Broth (TSB) were made up as per the manufacturer’s protocols. Sloppy agar consisted of 7 g/L of agar and 30 g/L of TSB. Before inoculating the pathogen into the sloppy agar, it must be melted. This was achieved by heating the agar at 105 °C for 8 min.

ESKAPE pathogens were grown overnight in 10 mL TSB cultures at 37 °C with shaking at 220 rpm. Fifty microliters of each ESKAPE overnight culture was inoculated into 100 mL of melted sloppy agar, mixed well, and overlaid onto assay plates.

### 4.3. Isolation of Bacteria from Soil Samples

For each respective soil sample, 5 g from each of the duplicates was dissolved in 10 mL of Ringer’s solution, and a dilution series was made up to 10^−6^. Point one of a milliliter was taken from the 10^−3^, 10^−4^, 10^−5^, and 10^−6^ dilutions and spread-plated onto four different solid media: SEA, SEA with cycloheximide, *Streptomyces* agar, and SCN agar; and incubated at 25 °C for 72 h. Individual colonies, particularly those that resembled the morphology of filamentous bacteria, those that exhibited pigmentation (as this is indicative of potential secondary metabolite production), and/or appeared to have secreted substance(s) into the environment surrounding the colony, were selected from these spread plates and were plated onto patch plates (of the same media type), where each patch plate consisted of 16 individual colonies. Thirteen unique patch plates, containing 208 colonies in total, were generated using this method. The 21 samples from an extant bank of antibiotic-producing garden soil isolates were also plated onto TSA patch plates for antibiotic production screening.

### 4.4. Antibiotic Profiling against ESKAPE Pathogens

Six replicates of each patch plate generated as described in Section 4.3 were plated on both TSA and garden soil SEA and subsequently incubated for 24 and 48 h, respectively, at 25 °C. One of the six replicate plates from each patch plate was overlayed with one of each of the six ESKAPE pathogens and incubated overnight at 30 °C. Zones of clearance surrounding individual colonies were deemed indicative of potential antibiotic production, and the diameters of colonies with such zones were measured. These isolates entered the next round of screening, where they were grown as a pure culture on either or both TSA or SEA, depending on which medium the zone of clearance had been observed and again overlaid with the ESKAPE pathogen to which potential antibiotic production had been observed. Isolates exhibiting antibiotic production activity were also plated in triplicate, with six replicates of each isolate, on a 20 mM D-GlcNAc SEA medium and incubated at 25 °C for 72 h. These replicates were overlaid with one of each of the six ESKAPE pathogens and incubated overnight at 30 °C.

### 4.5. 16S rRNA Gene Sequencing

The isolates exhibiting potential antibiotic activity were grown overnight in TSB at 25 °C with shaking at 220 rpm. One point five milliliter volumes of culture were subjected to DNA extraction using a GenElute microbial DNA extraction kit (Sigma-Aldrich), according to the manufacturer’s instructions. Quality and concentration were assessed by standard UV spectrophotometry. PCR amplification of the 16S gene was carried out where 31.5 µL of dH_2_O, 1 µL of dimethyl sulfoxide, 5 µL of 10× PCR buffer, 2 µL of dNTPs (2.5 mm stock), 3 µL of MgCl_2_ (25 mM stock), 1 µL of 27F forward primer (5′AGAGTTTGATCCTGGCTCAG) (10 mol/µL), 1 µL of 1492R reverse primer (5′GGTTACCTTGTTACGACTT) (10 mol/µL), and 0.5 µL of BioTaq (Bioline) polymerase were added per reaction with 5 µL of the target DNA for amplification. The thermocycling conditions used for the PCR reaction were 94 °C for 1 min, followed by 35 cycles of 94 °C for 30 s, 52 °C for 30 s, and 72 °C for 1 min, with a final extension step of 72 °C for 10 min in a DNA Engine^®^ Peltier PTC-200 Thermocycler (Bio-Rad, Hercules, CA, USA). PCR products were visualized using a 1% *w*/*v* Tris-Acetate-EDTA (TAE) buffer gel electrophoresis to ensure that PCR amplification had occurred successfully. PCR products were then purified for sequencing the QIAquick PCR Purification Kit (QIAGEN, Hilden, Germany, catalog number 28104), as per the manufacturer’s protocol. Purified PCR products were sent for sequencing at Eurofins Scientific. Sequencing results were inputted into BLASTn to identify isolates to the genus and potential species level based on >98% sequence identities. Future work is required to confirm species-level associations [28]. Individual 16S rRNA sequences can be retrieved from the Genbank nucleotide database using the accession numbers PQ428907–PQ428921.

## Figures and Tables

**Table 1:** Identity, isolation media, and ESKAPE pathogen antibiosis profiles of soil isolates.

Species	Isolation and Screening Media ^1^	ESKAPE_2_ Inhibition ^2^
*Streptomyces atratus*	SCN Agar	S
*Streptomyces atratus*	SCN Agar	S
*Paenibcillus* sp. Strain Y2	SCN Agar	S
*Pseudomonas azotoformans*	SEA	S, K, A
*Pseudomonas jessenii*	SEA + cycloheximide	S
*Streptomyces avidinii*	SEA	A
*Streptomyces goshikiensis*	SEA	K
*Paenibacillus polymyxa*	SEA	E, S, K, P
*Paenibacillus polymyxa*	SEA	E, S, K, P
*Streptomyces sporovareus*	SEA	E, S, K, A, P
*Paenibacillus peoriae*	SEA	E, S, K, A, P, E_2_
*Paenibacillus peoriae*	SEA	E, S, K, A, P, E_2_
*Paenibacillus peoriae*	SEA	E, S, K, A, P, E_2_
*Streptomyces* sp.	SEA	E
*Streptomyces/Kitosatospora* sp.	SEA	S, E

^1^ SCN = Starch-Casein-Nitrate, SEA = Soil extract agar. ^2^ ESKAPE_2_ pathogens: E = *E. faecium* NCIMB 11508; S = *S. aureus* N670 949, K = *K. pneumoniae* NCIMB 13218; A = *A. johnsonii* DSM 6963, P = *P. aeruginosa* PA01; E_2_ = *E. aerogenes* NCIMB 101P2.

**Table 2:** Antimicrobial activity induction on TSA.

Isolate	*E. faecium* NCIMB 11508	*S. aureus* N 670949	*K. pneumoniae* NCIMB 13218	*A. johnsonii* DSM 6963	*P. aeruginosa* PA01	*E. aerogenes* NCIMB 101P2
*Streptomyces atratus*	-	-	-	-	-	-
*Streptomyces atratus*	-	-	-	-	-	-
*Paenibacillus* sp. Strain Y2	-	1.2 ± 0 cm	1.7 ± 0 cm	2.1 ± 1 cm	-	-
*Pseudomonas azotoformans*	-	-	-	-	-	-
*Pseudomonas jensenii*	2.9 ± 0.2 cm	1.55 ± 0.7 cm	-	-	-	1.5 ± 0.4 cm
*Streptomyces avidnii*	-	-	-	-	-	-
*Streptomyces goshikiensis*	-	-	-	-	-	-
*Paenibacillus polymyxa*	-	0.95 ± 0.1 cm	1.5 ± 0.6 cm	-	1.45 ± 0.9 cm	1.75 ± 0.1 cm
*Paenibacillus polymyxa*	-	0.65 ± 0.1 cm	1.25 ± 1.7 cm	-	1.3 ± 0.2 cm	1.35 ± 0.3 cm
*Streptomyces spororaveus*	-	-	-	-	-	-
*Paenibacillus peoriae*	1 cm	1.35 ± 0.7 cm	1.45 ± 0.3 cm	1.35 ± 0.7 cm	1.8 ± 0.2 cm	3 ± 0.7 cm
*Paenibacillus peoriae*	-	1 ± 0.2 cm	1.8 cm	0.9 ± 0.2 cm	-	2.05 ± 0.5 cm
*Paenibacillus peoriae*	-	1.0 ± 0.4 cm	1.6 ± 0.2 cm	1.3 ± 0.2 cm	1.3 ± 0.4 cm	2.3 ± 3.0 cm
*Streptomyces* sp.	0.5 ± 0.4 cm	0.85 ± 0.3 cm	-	-	-	2.25 ± 2.5 cm
*Streptomyces/Kitosatospora* sp.	-	-	-	-	-	-

(-) indicates no antibiosis observed.

**Table 3:** Antimicrobial activity induction on SEA.

Isolate	*E. faecium* NCIMB 11508	*S. aureus* N 670949	*K. pneumoniae* NCIMB 13218	*A. johnsonii* DSM 6963	*P. aeruginosa* PA01	*E. aerogenes* NCIMB 101P2
*Streptomyces atratus*	-	0.8 ± 0.3 cm ^a^	-	-	-	-
*Streptomyces atratus*	-	0.6 ± 0.6 cm ^a^	-	-	-	-
*Paenibacillus* sp. Strain Y2	-	- ^b^	- ^b^	- ^b^	-	-
*Pseudomonas azotoformans*	-	1.35 ± 0.3 cm ^a^			-	-
*Pseudomonas jensenii*	- ^b^	- ^b^	-	-	-	1.7 ± 0.4 cm
*Streptomyces avidnii*	-	-	-	0.55 ± 0.1 cm ^a^	-	-
*Streptomyces goshikiensis*	-	-	0.5 cm ^a^	-	-	-
*Paenibacillus polymyxa*	-	0.85 ± 0.7 cm ^c^	0.75 ± 0.1 cm ^c^	0.65 ± 0.1 cm ^a^	0.7 ± 0 cm ^c^	1.6 ± 0.4 cm
*Paenibacillus polymyxa*	-	- ^b^	0.9 ± 0.8 cm	0.7 ± 0.2 cm ^a^	- ^b^	- ^b^
*Streptomyces spororaveus*	-	0.8 ± 0.3 cm ^a^	-	-	-	-
*Paenibacillus peoria*	0.8 ± 0.4 cm	1.45 ± 0.3 cm	0.65 ± 0.1 cm ^c^	0.9 ± 0.2 cm	0.55 ± 0.1 cm ^c^	0.75 ± 0.1 cm ^c^
*Paenibacillus peoria*	-	1.55 ± 0.1 cm	0.7 ± 0.2 cm ^c^	0.6 ± 0.6 cm ^c^	-	0.95 ± 0.3 cm ^c^
*Paenibacillus peoria*	-	1.65 ± 1.7 cm	0.7 ± 0.2 cm	0.85 ± 0.1 cm	0.45 ± 0.3 cm	1.25 ± 1.1 cm
*Streptomyces* sp.	-	-	-	-	-	-
*Streptomyces/Kitosatospora* sp.	3.3 ± 2.2 cm	4.35 ± 0.7 cm	-	-	-	-

(-) indicates no inhibition, ^a^ = inhibition observed on SEA only, ^b^ = loss of inhibition compared to TSA, ^c^ = reduced inhibition compared to TSA.

**Table 4:** Antimicrobial activity induction on SEA + GlcNAc.

Isolate	*E. faecium* NCIMB 11508	*S. aureus* N 670949	*K. pneumoniae* NCIMB 13218	*A. johnsonii* DSM 6963	*P. aeruginosa* PA01	*E. aerogenes* NCIMB 101P2
*Streptomyces atratus*	0.83 ± 0.2 cm ^a^	- ^b^	-	-	-	-
*Streptomyces atratus*	0.55 ± 0.3 cm ^a^	- ^b^	-	-	-	-
*Paenibacillus* sp. Strain Y2	-	-	-	-	-	-
*Pseudomonas azotoformans*	-	- ^b^	-	-	-	-
*Pseudomonas jensenii*	1.30 ± 0.2 cm	1.33 ± 0.1 cm	-	-	-	1.90 ± 0.2 cm
*Streptomyces avidnii*	0.73 ± 0.1 cm ^a^	0.83 ± 0.1 cm ^a^	0.77 ± 0.3 cm ^a^	0.67 ± 0.1 cm	-	1.03 ± 0.3 cm ^a^
*Streptomyces goshikiensis*	-	-	- ^b^	-	-	0.50 ± 0 cm ^a^
*Paenibacillus polymyxa*	-	0.63 ± 0.1 cm	0.87 ± 0.8 cm	0.75 ± 0.5 cm	- ^b^	2.30 ± 0.1 cm ^d^
*Paenibacillus polymyxa*		0.90 ± 0.6 cm ^a^	0.83 ± 0.1 cm	0.50 ± 0.3 cm	-	0.90 ± 0.2 cm ^a^
*Streptomyces spororaveus*	1.07 ± 0.5 cm ^a^	1.23 ± 0.3 cm	-	-	-	-
*Paenibacillus peoria*	- ^b^	0.83 ± 0.2 cm ^c^	0.40 ± 0.2 cm	0.60 ± 0.2 cm	- ^b^	0.77 ± 0.1 cm
*Paenibacillus peoria*	-	0.77 ± 0.1 cm	0.73 ± 0.1 cm	0.57 ± 0.3 cm	-	0.80 ± 0.3 cm
*Paenibacillus peoria*	-	0.70 ± 0.2 cm	0.50 ± 0.2 cm	0.77 ± 1.0 cm	- ^b^	0.80 ± 0.2 cm
*Streptomyces* sp.	0.80 ± 0.2 cm ^a^	-	-	-	-	-
*Streptomyces/Kitosatospora* sp.	1.23 ± 0.5 cm	- ^b^	-	-	-	-

(-) indicates no inhibition, ^a^ = inhibition observed on SEA + GlcNAc media only, ^b^ = loss of inhibition compared to SEA, ^c^ = reduced inhibition compared to SEA, ^d^ = increased inhibition compared to SEA.

## Data Availability

The data presented in this study are available in the article.
